# Skatole (3-Methylindole) Is a Partial Aryl Hydrocarbon Receptor Agonist and Induces CYP1A1/2 and CYP1B1 Expression in Primary Human Hepatocytes

**DOI:** 10.1371/journal.pone.0154629

**Published:** 2016-05-03

**Authors:** Martin Krøyer Rasmussen, Patrick Balaguer, Bo Ekstrand, Martine Daujat-Chavanieu, Sabine Gerbal-Chaloin

**Affiliations:** 1 INSERM, U1183, Institute of Regenerative Medicine and Biotherapy, Montpellier, F-34290, France; 2 Montpellier University, UMR 1183, Montpellier, F-34203, France; 3 Department of Food Science, Aarhus University, Foulum, Denmark; 4 Institut de Recherche en Cancérologie de Montpellier, Montpellier, F-34298, France; 5 CHU Montpellier, Institute of Regenerative Medicine and Biotherapy, Montpellier, F-34290, France; French National Centre for Scientific Research, FRANCE

## Abstract

Skatole (3-methylindole) is a product of bacterial fermentation of tryptophan in the intestine. A significant amount of skatole can also be inhaled during cigarette smoking. Skatole is a pulmonary toxin that induces the expression of aryl hydrocarbon receptor (AhR) regulated genes, such as cytochrome P450 1A1 (CYP1A1), in human bronchial cells. The liver has a high metabolic capacity for skatole and is the first organ encountered by the absorbed skatole; however, the effect of skatole in the liver is unknown. Therefore, we investigated the impact of skatole on hepatic AhR activity and AhR-regulated gene expression. Using reporter gene assays, we showed that skatole activates AhR and that this is accompanied by an increase of *CYP1A1*, *CYP1A2* and *CYP1B1* expression in HepG2-C3 and primary human hepatocytes. Specific AhR antagonists and siRNA-mediated AhR silencing demonstrated that skatole-induced *CYP1A1* expression is dependent on AhR activation. The effect of skatole was reduced by blocking intrinsic cytochrome P450 activity and indole-3-carbinole, a known skatole metabolite, was a more potent inducer than skatole. Finally, skatole could reduce TCDD-induced *CYP1A1* expression, suggesting that skatole is a partial AhR agonist. In conclusion, our findings suggest that skatole and its metabolites affect liver homeostasis by modulating the AhR pathway.

## Introduction

Skatole (3-methylindole) is a product of the bacterial breakdown of tryptophan and is found in the intestine of humans and pigs [1]. Additionally, a significant amount of skatole can be inhaled during cigarette smoking [2]. In pigs, low hepatic clearance of skatole might be responsible for the off-flavor/odor of the meat from some sexually mature male pigs [3, 4]. Moreover, skatole can have pneumotoxic effects in several species, including humans, but not in pigs [1, 5]. This could depend on skatole metabolites produced by specific cytochrome P450 (CYP) isoforms, particularly CYP2F1 and CYP3A4 [6]. Moreover, skatole or its metabolites might induce DNA damage [7], inhibit lipid peroxidation [8] and decrease glutathione content [9].

The CYP1As are part of the phase I enzymes involved in the metabolism of several pharmacologic compounds, natural plant products, environmental pollutants and toxins [10]. Moreover, CYP1A enzymes convert specific pro-carcinogens into full carcinogens [11]. Human, mouse and porcine CYP1A also metabolize skatole [12–14]. Human CYP1A1 is mostly expressed in extrahepatic tissues, but can be induced in the liver. Conversely, CYP1A2 is predominantly expressed in liver, and weakly in intestine [15]. It is generally accepted that CYP1As are controlled by the aryl hydrocarbon receptor (AhR) pathway. When activated, AhR dissociates from the cytosolic complex formed with HSP90, translocates into the nucleus where it heterodimerizes with AhR Nuclear Translocator (ARNT) and eventually binds to specific DNA sequences (Xenobiotic Responsive Elements, XRE) in the promoter region of its target genes, thus initiating gene transcription. AhR can be activated by several endogenous and exogenous compounds [16, 17], including tryptophan and its metabolites [18–20]. Skatole can activate AhR and initiate *CYP1A* transcription in primary human bronchial epithelial cells and colonic cell lines (Caco2) [21, 22]; however, it is unknown whether it can do it also in liver cells, especially primary cells that poses more metabolic activity compared to cell-lines.

To our knowledge most research on skatole physiopathological effects in humans has been focused on the respiratory tract, because of its importance as pneumotoxin. However, the liver could also be an important skatole target due to its strategic placement as the first organ encountered by skatole absorbed from the intestine before entering the circulatory system and due to its higher capacity to metabolize skatole compared to the lungs [23].

Here, we tested the hypothesis that skatole regulates the expression of CYP enzymes by modulating AhR activity. To this aim, we investigated the ability of skatole to activate AhR in reporter gene assays. Moreover, we incubated HepG2-C3 cells (a human hepatoblastoma cell line) and primary human hepatocytes (PHHs) with skatole and its metabolite indole-3-carbinole (I3C) and evaluated their effect on CYP/AhR gene and protein expression. We also examined the combined effect of the prototypical AhR activator TCDD and skatole or I3C. We found that skatole is a weak activator and a partial agonist of AhR and that its activity depends on its CYP-mediated conversion into more active metabolites.

## Material and Methods

### Chemicals

TCDD (2,3,7,8-tetrachlorodibenzo-p-dioxine), skatole (3-methylindole), indole-3-carbinol (I3C), 1-aminobenzotriazole (ABT) and actinomycin D were from Sigma-Aldrich (Saint-Louis, MO, US). CH-223191 (2-Methyl-2H-pyrazole-3-carboxylic acid-(2-methyl-4-o-tolyl-azophenyl)-amide, an antagonist of TCDD-mediated AhR activation [24, 25], was from Calbiochem (Merck KGaA, Damstadt, Germany).

### Cell culture

HepG2-C3 cells (ATCC) were cultured as recommended in Minimum Essential Medium (MEM) supplemented with 10% fetal calf serum (FCS) and 2 mM glutamine, 1 mM sodium pyruvate, 1% non-essential amino acids, 100 units/mL of penicillin and 100 μg/mL of streptomycin, in a 5% CO_2_ humidified atmosphere at 37°C. Cells were used at low passage number (<20) to ensure constitutive AhR expression.

The transfected AhR reporter cell lines HAhLH (stably transfected) and HepAhLH (transiently transfected) were obtained by transfecting HeLa and HepG2 cells, respectively, with the _5_(TnGCGTG)_3_-tata-luciferase-Luc-hygromycin plasmid in which the luciferase reporter gene is controlled by the XRE _5_(TnGCGTG)_3_. HAhLH and HepAhLH cells were grown in Dulbecco’s Modified Eagle’s Medium F12 (DMEM/F12) with phenol red, supplemented with 5% FCS, 100 units/mL of penicillin and 100 μg/mL of streptomycin and 0.25 mg/ml hygromycin in 5% CO_2_ humidified atmosphere at 37°C.

### Human liver samples and preparation of primary human hepatocytes

Liver samples were obtained from resections performed in adult patients for medical reasons unrelated to our research program or from donors when the liver was considered unsuitable for organ transplantation. Regarding livers from non-transplantable organ donor, the informed consent of the donor's family was obtained by the Service de la Coordination Hospitalière (CHU Montpellier). The use of liver lobectomies resected for medical reasons, or livers of organ donors not suitable for transplantation for hepatocytes isolation for research purposes has been approved by French National Ethics Committees and legal instance, and by the French Graft Institute “Agence de Biomedecine”, respectively. All human liver samples entering the laboratory Inserm U1183 are collected after patients have signed informed consent in agreement with ethics procedures and adequate authorisations obtained from the Ministère de l’Enseignement Supérieur et de la Recherche, (reference: MESR DC-2008-531). The clinical characteristics of the liver donors are given in Table 1. PHHs were prepared as described previously and cultured in collagen-coated dishes at a density of 1.7 x 10^5^ cells/cm^2^ in ISOM medium [26, 27]. At day 3 post-isolation, cells were incubated with the different compounds or with equal amounts of vehicle (DMSO: final concentration 0.1%) (controls). All treatments were done in duplicates.

**Table 1:** Clinical characteristics of the liver donors.

Liver identification	Sex	Age	Pathology
PHH390	M	35	Organ donor
PHH391	F	29	Cystadenoma
PHH396	M	59	Organ donor
PHH397	M	53	Organ donor
PHH400	M	60	Organ donor
PHH401	M	76	Hepatocellular carcinoma

### Transfection with siRNA

Adherent PHHs were transfected with 20 nM non-targeting siRNA or siRNA specific for *AhR* (Dharmacon, Lafayette, CO) using Lipofectamine RNAiMAX (Life Technology) at day 1 and day 3 post-seeding. At day 5 post-seeding, PHHs were incubated with the indicated compounds for 24 h. All treatments were done in duplicates.

### RNA isolation and PCR

After extraction with Trizol reagent (Invitrogen), 500 ng of total RNA was reverse-transcribed using a random hexaprimer and the MMLV Reverse Transcriptase Kit (Invitrogen). Quantitative PCR was performed using the Roche SYBER Green reagent and a LightCycler 480 apparatus (Roche Diagnostic, Meylan, France) with the following program: one step at 95°C for 10 min, 40 cycles of denaturation at 95°C for 10 sec, annealing at 65°C for 15 sec and elongation at 72°C for 15 sec. The amplification specificity was evaluated by determining the product melting curve. Results are expressed as indicated in the figure legends. Primer sequences are given in Table 2.

**Table 2:** Primer sequences.

Gene Name	Forward primer	Reverse primer
AhR	ATCAGTGCCAGCCAGAACCTC	AGGTCTGGCTTCTGACGGATG
CYP1A1	TCCGGGACATCACAGACAGC	ACCCTGGGGTTCATCACCAA
CYP1A2	CATCCCCCACAGCACAACAA	TCCCACTTGGCCAGGACTTC
CYP1B1	GCCACTATCACTGACATCTTCGG	CACGACCTGATCCAATTCTGCC
Actin	TGGGCATGGGTCAGAAGGAT	TCCATCACGATGCCAGTGGT
RPLP0	TCGACAATGGCAGCATCTAC	GCCTTGACCTTTTCAGCAAG

The relative mRNA expression was normalized to the expression of β-actin and ribosomal protein large P0 (RPLP0), as recommended by Vandesompele et al [28]. The Ct-values for β-actin and RPLP0 were not affected by any of the treatments.

### Reporter gene assay

HepG2-C3 cells were transfected in suspension with 200 ng of pTXINV-XRE-luc reporter plasmid [29] and 20 ng of pTK-luc Renilla control vector in Opti-MEM I medium (Invitrogen) using Lipofectamine 2000 (Life Technology) for 24 h, according to the manufacturer’s instructions. The medium was then renewed and cells were pre-incubated or not with 3 μM CH223191 for 30 min and then with skatole, TCDD or DMSO, as indicated. Dual luciferase assays were performed according to the manufacturer’s specifications (Promega, Madison, WI) using a Mithras LB940 apparatus (Berthold Technologies). Results from treated samples were expressed relative to the mean value of the control samples (arbitrarily set to 1). All treatments were done in triplicate and repeated in at least one independent experiment.

HAhLH and HepAhLH reporter cells were seeded at a density of 25,000 cells per well in 96-well white opaque tissue culture plates with 150 μl culture medium. Cells were incubated with different concentrations of skatole or I3C (between 0.1 and 100 μM), or TCDD (between 0.01 nM and 1 μM) for 8 h. At the end of the incubation time, the medium with the test compounds was removed and replaced with culture medium containing 0.3 mM luciferin. At this concentration, luciferin diffuses in the cells and produces a stable luminescent signal after five min. Luminescence was measured in intact living cells for 2 sec using a luminometer. Tests were performed in quadruplicate for each concentration. Results were expressed as the percentage of the maximal luciferase activity (100%) obtained in the presence of 100 nM TCDD.

### Western blotting

Total protein extracts were prepared using RIPA buffer (Sigma-Aldrich) supplemented with a protease inhibitor cocktail (Santa Cruz Biotechnology, Santa Cruz, CA). The protein concentration was determined by the bicinchoninic acid method, according to the manufacturer’s instructions (Pierce Chemical Co., Rockford, IL). Bovine serum albumin (Pierce Chemical Co.) was used as standard. Equal amounts of total proteins were separated on precast SDS-polyacrylamide gels (4–16%) (Bio-Rad, Marnes la Coquette, France), then transferred onto PVDF membranes (Bio-Rad). Membranes were incubated with mouse monoclonal anti-CYP1A1/2 (SC-53241, Santa Cruz) or goat polyclonal anti-actin (SC-1616, Santa Cruz) antibodies. Immunocomplexes were detected with horseradish peroxidase-conjugated mouse or goat secondary antibodies (Sigma) followed by enhanced chemiluminescence reaction (Millipore, Molsheim, France). Chemiluminescence was monitored using a ChemiDoc-XRS^+^ apparatus (Bio-Rad Laboratories) and quantified using the Image Lab software (version 4.1)

### Assay for CYP1A activity

The relative CYP1A activity was assessed using the P450-Glo^™^ CYP1A1 Assay (Promega), with luciferin-CEE as substrate. All obtained activity values were normalized to the cell viability assessed using the CellTiter-Glo^™^ assay (Promega), according to the manufacturer’s instruction.

### Statistics

All statistical tests were performed using the SigmaPlot software. For comparison of two groups, the Students unpaired t-test was used, while for comparison of multiple groups, ANOVA with Tukey’s post hoc test was used. If data were no normally distributed, the ANOVA test were executed on log10-transformed data. In all tests, p < 0.05 was considered as significant.

## Results

### Skatole activates AhR in reporter gene assays

**Fig 1:**
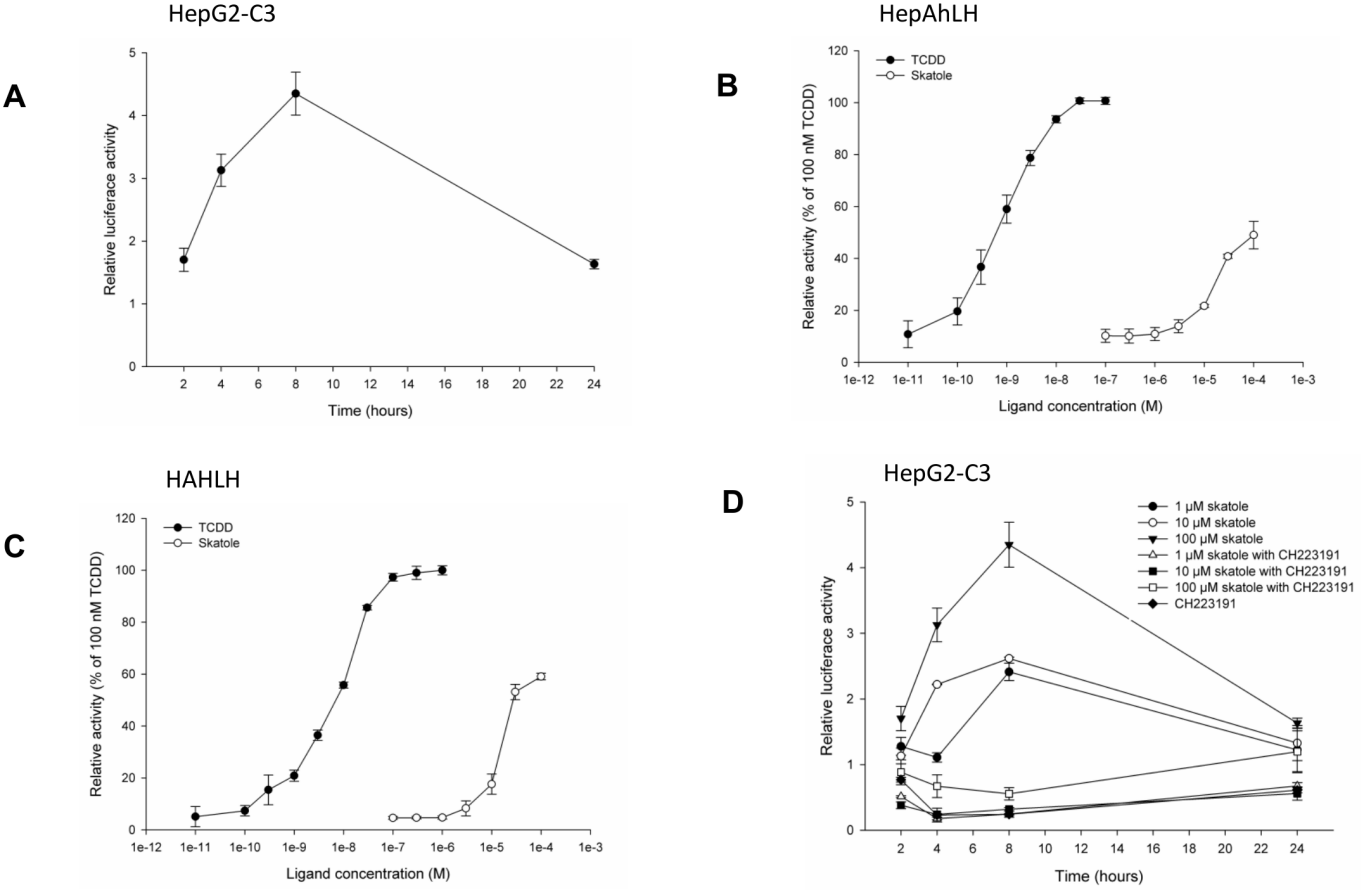
Skatole activates AhR. (A) Luciferase activity in HepG2-C3 cells transiently transfected with the pTXINV-XRE reporter plasmid and incubated with 100 μM skatole for 2, 4, 8 or 24 h (n = 3). Luciferase activity measured in HepAhLH (B) or HAhLH (C) cells, in which the luciferase reporter gene is controlled by the XRE _5_(TnGCGTG)_3,_ after incubation with skatole (from 1.10^−7^ M to 1.10^−4^ M) or TCDD (from 1.10^−11^ M to 1.10^−7^ M) for 8 h (n = 3). Luciferase activity was expressed as the percentage of the activity obtained by incubation with 1.10^−7^ M TCDD. (D) Relative luciferase activity measured in HepG2-C3 cells transfected with pTXINV-XRE and incubated with 1, 10 or 100μM skatole for 2, 4, 8 or 24 h, following 30 min pre-incubation with DMSO (control) or 3 μM CH223191 (a specific AhR antagonist) (n = 3). In Fig 1D, are all data points significantly different (p < 0.05) form its time-matched CH 223191 treated counterpart, except for control and 100 μM skatole at time point 24 hours.

To test whether skatole activates AhR, hepatic and non-hepatic reporter cell lines were used. First, HepG2-C3 cells were transiently transfected with the pTXINV-XRE-luc reporter plasmid and incubated with 100 μM skatole for 24 h to determine a time-response curve (Fig 1A). Luciferase activity progressively increased, but after 8 h of incubation started to decrease back to control levels. Then, hepatic HepG2 (HepAhLH) and non-hepatic HeLa (HAhLH) reporter cells (stably transfected with the _5_(TnGCGTG)_3_-tata-luciferase-Luc-hygromycin plasmid) were incubated with increasing concentrations of skatole or TCDD (the prototypical AhR activator) for 8 h. Luciferase activity increased in a dose-dependent manner in both cell lines (Fig 1B and 1C) and with both compounds, although the TCDD effect was significantly stronger than that of skatole (Fig 1B and 1C). The observed increase in luciferase activity was reduced when cells were pre-incubated with CH223191 (a known inhibitor of TCDD-mediated AhR-dependent transcription [24]) for 30 min before stimulation with skatole or TCDD (Fig 1D). These results indicate that skatole activates AhR in liver cells.

### Skatole increases the expression of CYP1A1, CYP1A2 and CYP1B1 in an AhR-dependent manner

**Fig 2:**
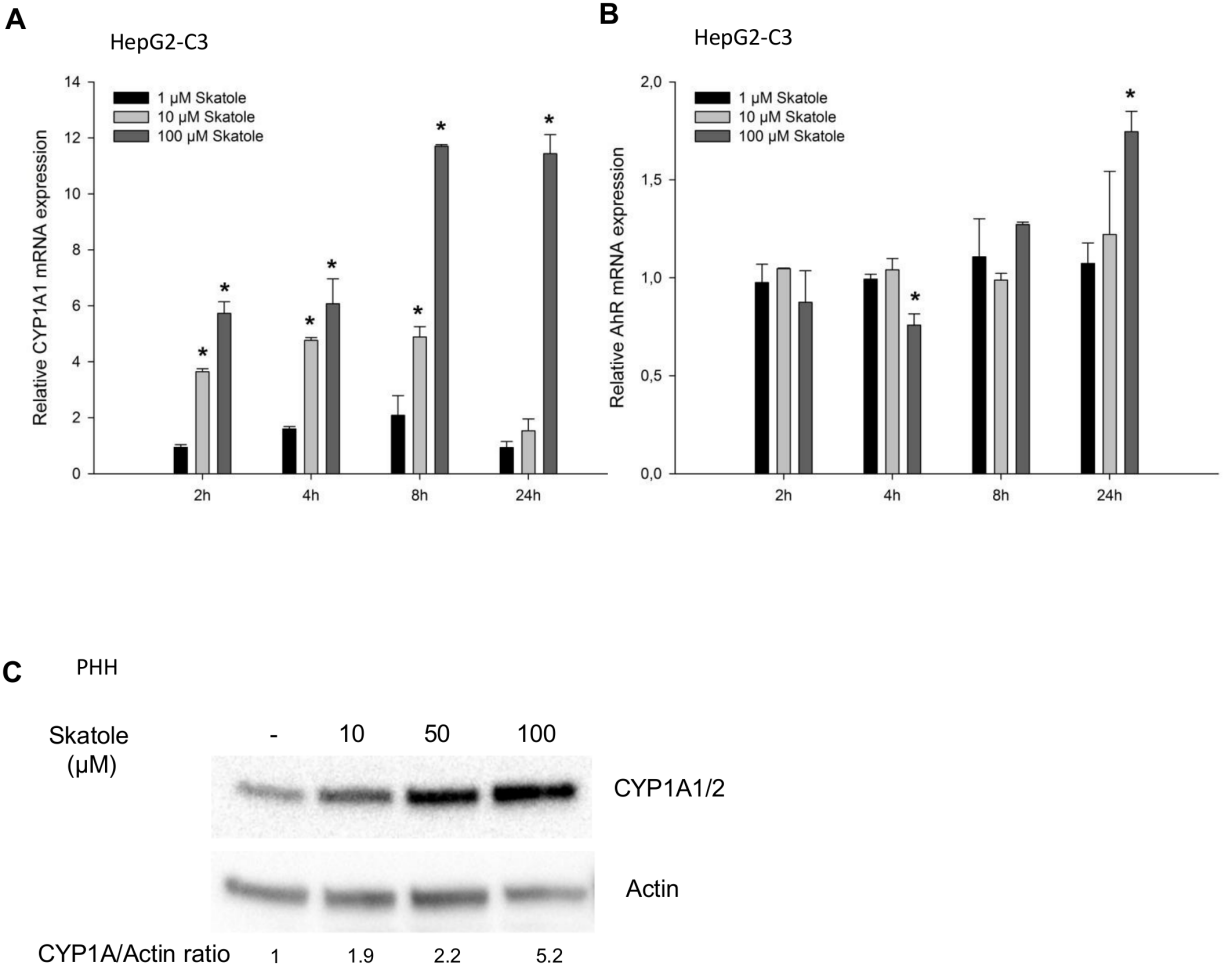
Skatole increases CYP1A1 expression in HepG2-C3 cells and PHHs. RT-qPCR analysis of *CYP1A1* (A) and *AhR* (B) mRNA expression following incubation of HepG2-C3 cells with 1, 10 or 100 μM skatole for 2, 4, 8 or 24 h (n = 3). (C) CYP1A and actin protein expression in PHHs (Donor #400) after incubation with 10, 50 or 100 μM for 24 h. * Significantly different from time-matched control cells (no treatment) (Student’s t–test; p < 0.001).

To further test whether skatole is an AhR activator, HepG2-C3 cells were incubated with 1, 10 or 100 μM skatole for 2 to 24 h and the effect on the transcription of AhR-regulated genes was determined. After 2 and 8 h, *CYP1A1* mRNA expression was significantly increased in cells incubated with 10 or 100 μM skatole compared to DMSO-treated cells (Fig 2A). Conversely, after 24 h, *CYP1A1* expression was further increased only in cells incubated with 100μM skatole. Incubation with 1 μM skatole had no effect on *CYP1A1* mRNA expression. The induction of *CYP1A1* mRNA was not accompanied by major changes in AhR mRNA expression (Fig 2B).

The effect of skatole on CYP1A1/2 and CYP1B1 expression was then evaluated also in PHHs, which are considered the gold standard model to assess human liver metabolism. Similar to what was observed in HepG2-C3 cells, incubation with skatole for 8 and 24 h increased *CYP1A1*, *CYP1A2* and *CYP1B1*, but not *AhR* mRNA expression in all tested PHHs (n = 5 donors) (Table 3). Although the mRNA induction upon incubation with skatole greatly varied among PHHs, reflecting the known inter-donor variability, no correlation between the clinical characteristics of the liver donor and the level of *CYP* induction in PHHs was observed. Skatole also increased CYP1A1/2 protein expression in PHHs (Fig 2C).

**Table 3:** *CYP1A1*, *CYP1A2*, *CYP1B*1 and *AhR* mRNA expression in PHHs (n = 5) incubated with 1, 10, 100 μM skatole or 10 nM TCDD for 8 or 24 hours.

	Incubation time	1 μM skatole	10 μM skatole	100 μM skatole	10 nM TCDD
*CYP1A1*	8 hours	1.1 ± 0.5 *(0*.*5–1*.*8)*	4.1 ± 2.8 *(0*.*8–8*.*4)*	10.6 ± 6.5^b,^* *(1*.*9–19*.*9)*	52.0 ± 30.2* *(16*.*7–73*.*6)*
	24 hours	1.2 ± 1.1 *(0*.*4–3*.*3)*	3.1 ± 2.2 *(1*.*3–7*.*5)*	26.0 ± 22.6*^,^ **^,^ *** *(4*.*6–66*.*1)*	548.7 ± 445.8* *(45*.*3–1275*.*6)*
*CYP1A2*	8 hours	1.1 ± 0.3 *(0*.*7–1*.*4)*	2.2 ± 0.8 *(1*.*0–3*.*3)*	4.2 ± 2.6*^,^ ** *(1*.*1–8*.*7)*	41.4 ± 52.9 *(6*,*3–145*.*5)*
	24 hours	1.1 ± 0.6 *(0*.*6–2*.*3)*	2.0 ± 0.7 *(1*.*2–3*.*4)*	10.4 ± 8.9*^,^ **^,^ *** *(2*.*5–27*.*3)*	410.6 ± 296.9* *(201*.*5–935*.*9)*
*CYP1B1*	8 hours	1.0 ± 0.5 *(0*.*4–1*.*6)*	3.0 ± 2.1 *(0*.*5–5*.*5)*	7.2 ± 4.1*^,^ ** *(2*.*5–14*.*7)*	56.9 ± 82.3 *(11*.*0–221*.*0)*
	24 hours	1.0 ± 0.3 *(0*.*5–1*.*2)*	1.1 ± 0.1 *(1*.*0–1*.*3)*	2.6 ± 0.7*^,^ **^,^ *** *(2*.*0–3*.*9)*	318.9 ± 404.7 *(46*.*4–1119*.*3)*
*AhR*	8 hours	1.0 ± 0.1 *(0*.*8–1*.*1)*	0.9 ± 0.2 *(0*.*8–1*.*3)*	0.8 ± 0.1 *(0*.*7–1*.*0)*	0.8 ± 0.2* *(0*.*5–1*.*0)*
	24 hours	1.0 ± 0.2 *(0*.*7–1*.*2)*	0.9 ± 0.1 *(0*.*8–1*.*0)*	1.0 ± 0.1 *(0*.*9–1*.*1)*	0.8 ± 0.3 *(0*.*5–1*.*2)*

All values (mean ± SD; Data range within brackets) are expressed as fold change compared to control PHHs (only DMSO).

* significantly different from control (DMSO treated PHH)

** significantly difference between samples treated with 1 μM and 100 μM skatole

*** significantly difference between samples treated with 10 μM and 100 μM skatole

**Fig 3:**
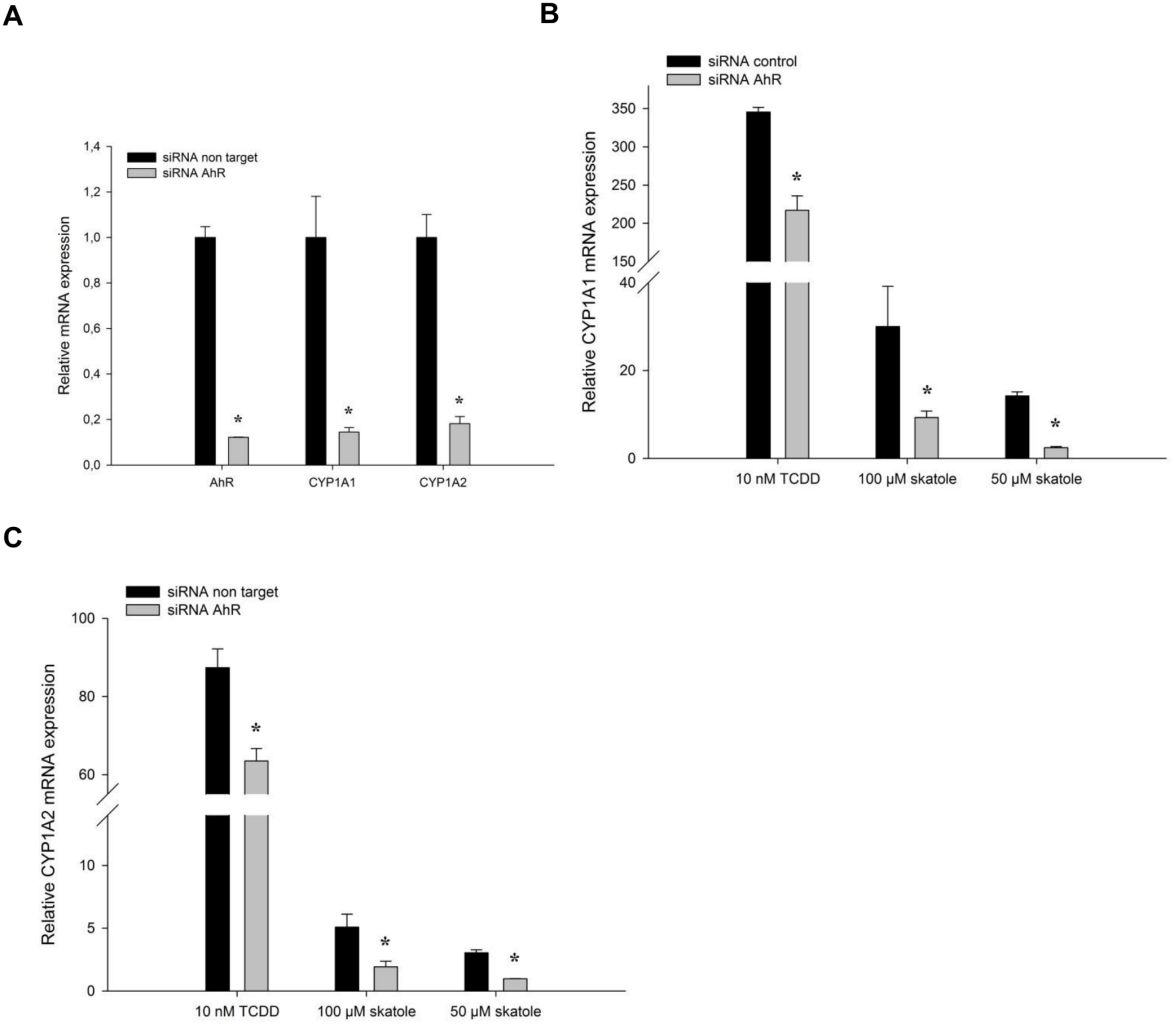
AhR is required for skatole-mediated CYP1A1 and CYP1A2 up-regulation. (A) RT-qPCR analysis of *AhR*, *CYP1A1* and *CYP1A2* mRNA expression in PHHs (Donor #391) after siRNA-mediated *AhR* down-regulation. (B) RT-qPCR analysis of *CYP1A1* (B) and *CYP1A2*(C) mRNA expression in PHHs after siRNA-mediated *AhR* down-regulation and incubation with 50 or 100 μM skatole, or 10 nM TCDD. * significantly different from cells transfected with non-target siRNA.

Finally, AhR activation by skatole was confirmed by using PHHs in which AhR was silenced by siRNA. The basal mRNA expression of *AhR* and its target genes *CYP1A1* and *CYP1A2* was reduced by approximatively 90% in silenced PHHs compared with PHHs transfected with non-targeting siRNA (Fig 3A). Moreover, the increase in *CYP1A1* and *CYP1A2* mRNA expression upon incubation with skatole or TCDD was reduced in *AhR*-silenced PHHs compared with PHHs transfected with non-targeting siRNA (Fig 3B and 3C).

### Skatole is an AhR partial agonist

**Fig 4:**
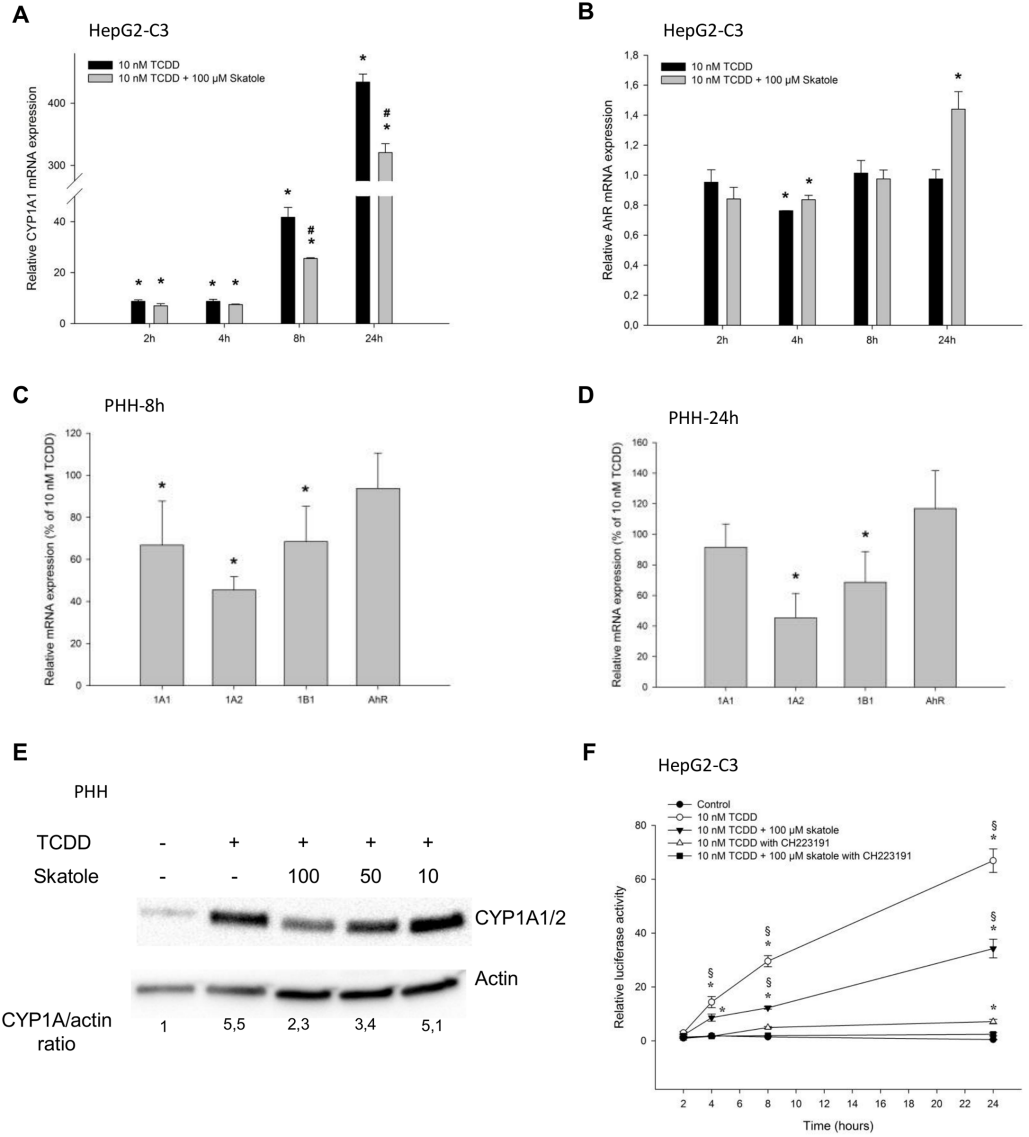
Skatole reduces TCDD-induced AhR activity and *CYP1A1* expression. RT-qPCR analysis of (A) *CYP1A1* and (B) *AhR* mRNA expression following incubation of HepG2-C3A cells with 10 nM TCDD in the presence or not of 100 μM skatole for 2, 4, 8 or 24 h (n = 3). RT-qPCR analysis of *CYP1A1*, *CYP1A2*, *CYP1B1* and *AhR* mRNA expression in PHHs treated for 8 (C) or 24 h (D) with TCDD in the presence or not of 100 μM skatole (n = 5). Results are expressed as the percentage of the induction observed with 10 nM TCDD. (E) CYP1A and actin protein expression in PHHs after incubation with 10 nM TCDD in the presence or not of 10, 50 or 100 μM skatole for 24 h (Donor #401). (F) Luciferase activity in HepG2-C3 cells transfected with pTXINV-XRE and incubated with TCDD alone or with skatole, in the presence or not of 3 μM CH223191 for 2, 4, 8 or 24 h (n = 3). * Significantly different from time-matched control cells (DMSO); # significantly different from time-matched TCDD treated cells (Student’s t–test; p < 0.01).

The finding that skatole could not elicit the maximum possible response obtained with the full agonist TCDD in cell reporter assays suggests that skatole is an AhR partial agonist. This is supported by the pronounced differences in the extent of *CYP1A1/2* and *CYP1B1* induction observed in PHHs upon incubation with TCDD or skatole (Table 3). In the presence of a full agonist, a partial agonist will act as an antagonist, competing with the full agonist and thereby reducing its ability to produce its maximum effect. To test this, HepG2-C3 cells and PHHs were incubated with TCDD (full agonist) and skatole on their own or in combination. TCDD alone strongly increased *CYP1A1* and *CYP1A2* mRNA expression in both HepG2-C3 cells and PHHs (Table 3 and Fig 4A and 4B). When HepG2-C3 cells were co-incubated with 10 nM TCDD and 100 μM skatole, *CYP1A1* mRNA induction was significantly reduced compared with cells incubated with TCDD alone (Fig 4A), whereas *AhR* mRNA expression was minor affected (Fig 4B). Similarly, in PHHs, *CYP1A1*, *CYP1A2* and *CYP1B1* up-regulation was significantly reduced after 8 h of co-incubation with TCDD and skatole compared to TCDD-treated cells (Fig 4C). On the other hand, after 24 h of co-incubation, only *CYP1A2* and *CYP1B1* expression were affected (Fig 4D). Co-incubation with skatole and TCDD also reduced CYP1A1/2 protein up-regulation, in comparison with TCDD alone, both in HepG2C3 cells (data not shown) and in PHHs (Fig 4E).

To test whether the CYP expression decrease observed upon co-incubation with TCDD and skatole relative to TCDD alone was due to decreased AhR activation, HepG2-C3 cells transiently transfected with the pTXINV-XRE-luc reporter plasmid were incubated with TCDD alone, or with skatole and/or CH-223191 (inhibitor of TCDD-mediated AhR-dependent transcription) and luciferase activity was measured at different time points (Fig 4F). Incubation with TCDD alone or with skatole increased luciferase activity over time, although the increase was significantly lower in cells co-treated with TCDD and skatole compared to TCDD alone. Addition of CH-223191 reduced luciferase activity almost to control (no treatment) level.

### Effect of skatole metabolites on CYP1A expression

**Fig 5:**
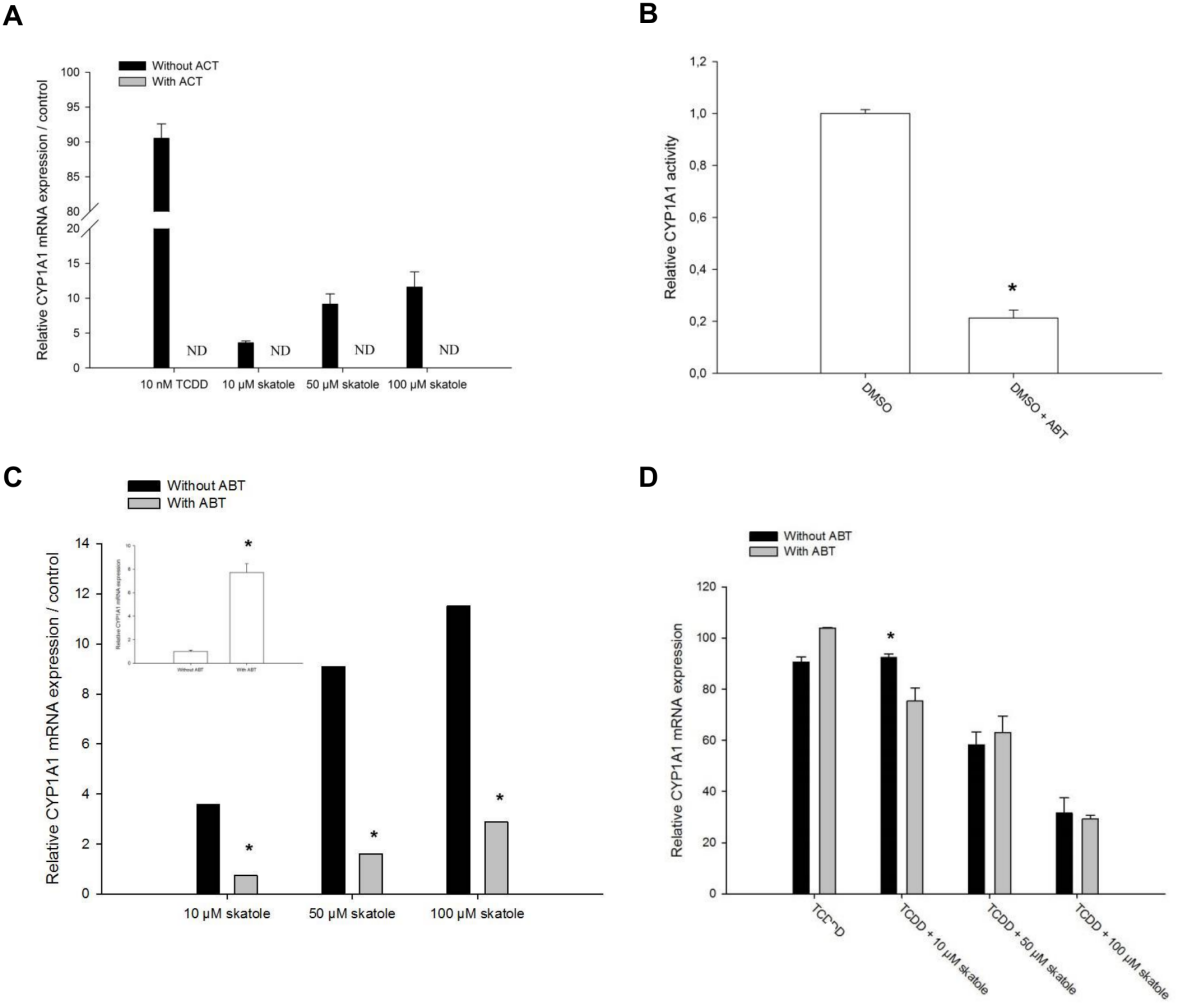
Effect of skatole metabolism on CYP1A induction in HepG2-C3 cells. (A) RT-qPCR analysis of *CYP1A1* mRNA expression in HepG2-C3 cells following incubation with 4 μM actinomycin D for 1 h and incubation with 10 nM TCDD or 10, 50 or 100 μM skatole for 8 h (n = 3). (B) Relative CYP1A1 activity following incubation with 1 mM ABT for 1 h (n = 3). (C) RT-qPCR analysis of *CYP1A1* mRNA expression in HepG2-C3 cells that were pre-incubated or not with 1 mM ABT for 1 h before incubation with 10, 50 or 100μM skatole for 8 h (n = 3). Insert shows *CYP1A1* mRNA expression in HepG2-C3 cells after incubation or not with ABT. (D) RT-qPCR analysis of *CYP1A1* mRNA expression in HepG2-C3 cells that were pre-incubated or not with 1 mM ABT for 1 h before incubation with 10 nM TCDD alone or together with 10, 50 or 100μM skatole for 8 h (n = 3). * significantly different from control cells; § significantly different from its time-matched CH 223191 treated counterpart.

To explore the mechanism responsible for the observed increase in *CYP1A* expression in skatole- treated cells, HepG2-C3 cells were pre-incubated with 4 μM of actinomycin D, a known transcription inhibitor, for 1 h. Actinomycin D had no effect on cell viability (S1 Fig). Actinomycin D blocked the effect of both TCDD and skatole on *CYP1A1* expression (Fig 5A), without affecting the expression of the housekeeping genes (β-actin and RPLP0) (S2 Fig). The same was true for *CYP1A2* (S3 Fig). This demonstrates that *CYP1A* induction in response to skatole occurs at the transcriptional level.

Then, we tested whether skatoles effect was due to skatole directly or whether metabolic conversion by CYPs is required for full activity. To inhibit CYP activity, HepG2-C3 cells were incubated with 1 mM ABT (a specific inhibitor of animal CYPs) [9, 30] for 1 hour. This treatment reduced CYP1A1/1B1-dependent activity by approximatively 80% compared to DMSO-treated cells, as assessed by using the P450-Glo^™^ CYP1A1 assay (Fig 5B). Then, HepG2-C3 cells were pre-incubated with 1 mM ABT for 1 h before addition or not of skatole or TCDD. After 8 h, *CYP1A* mRNA expression was increased in cells incubated with ABT alone (insert Fig 5C). Conversely, after skatole addition, *CYP1A* mRNA expression was significantly reduced in cells pre-incubated with ABT compared to cells without ABT (Fig 5C), suggesting that skatole metabolism is required to obtain full induction. As expected, ABT had no effect on TCDD-induced CYP1A1 expression (Fig 5D), in agreement with the fact that TCDD is poorly metabolized and has a half-life of ~5 years in adults [31]. Moreover, ABT had no effect on skatole-mediated inhibition of *CYP1A1* induction by TCDD (Fig 5D). This suggests that the observed inhibitory effect on TCDD-induced CYP1A1 expression is caused by skatole and not by a metabolite.

**Fig 6:**
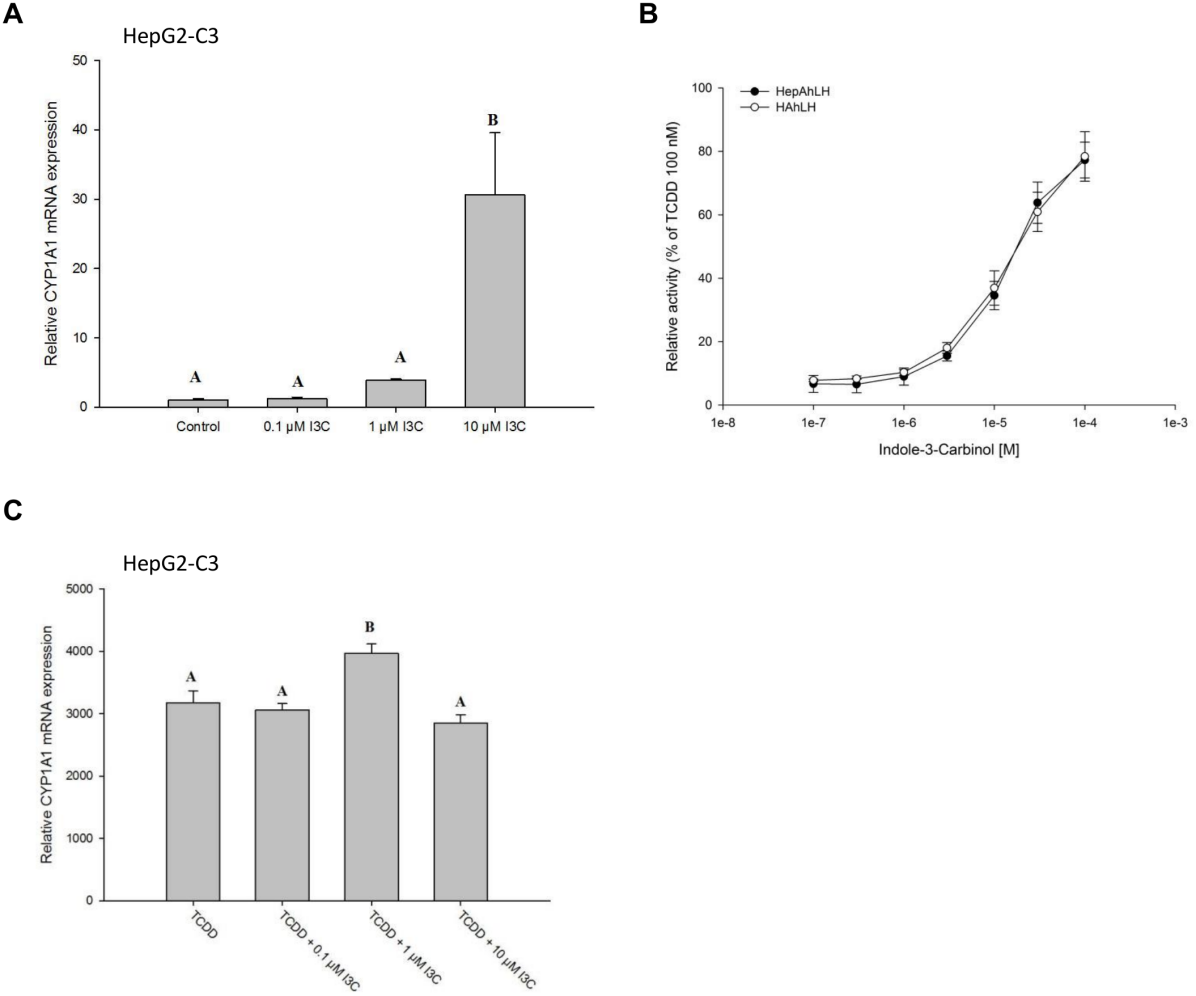
Indole-3-carbinol (I3C) induces CYP1A1 expression and activates AhR. (A) RT-qPCR analysis of *CYP1A1* mRNA expression in HepG2-C3 cells incubated with 0.1, 1 or 10 μM I3C for 24 h (n = 3). (B) Relative luciferase activity measured in HAhLH or HepAhLH cells incubated with I3C (from 1.10−^7^ M to 1.10−^4^ M) for 8 h (n = 3). (C) RT-qPCR analysis of *CYP1A1* mRNA expression in HepG2-C3 cells incubated with 10 nM TCDD alone or in the presence of 0.1, 1 or 10 μM I3C (n = 3). Bars not sharing subscription are significantly different (p < 0.05).

To investigate whether a skatole metabolite could be the main responsible for *CYP1A* mRNA up-regulation, HepG2-C3 cells were incubated with various concentrations of I3C, a commercially available skatole metabolite [14, 32, 33], for 24 h. I3C induced *CYP1A1* mRNA expression more potently than skatole. Indeed, 10 μM I3C increased *CYP1A1* mRNA expression by about 30-fold (relative to the DMSO control; Fig 6A) compared with the 2-fold increase observed with 10 μM skatole (Fig 2A). This was confirmed in reporter experiments performed in HAhLH and HepAhLH cells where I3C was a more potent activator of luciferase activity than skatole (Fig 6B; compare with Fig 1B and 1C). Conversely, I3C had no effect on TCDD-mediated *CYP1A1* mRNA expression up-regulation in HepG2-C3 cells (Fig 6C).

## Discussion

In humans, little is known about skatole effects on the expression of drug-metabolizing enzymes in the liver. Here, we show, using different cell models and PHHs, that skatole increases in a time- and dose-dependent manner the mRNA levels of *CYP1A1*, *CYP1A2* and *CYP1B1* genes, the expression which is controlled by AhR. Using reporter-gene assays and siRNA–mediated down-regulation of AhR expression, we demonstrate that skatole-induced *CYP1A* expression up-regulation is dependent on AhR activation. The increase in CYP mRNA expression after incubation with skatole was low (< 1/10) compared to the increase following treatment with TCDD, a full AhR activator. Moreover, skatole showed antagonistic activity towards TCDD-mediated AhR activation, suggesting that skatole is a weak activator and a partial agonist of AhR. Finally, we show that skatole effect is dependent on its metabolic conversion in the liver, suggesting that its metabolites (e.g., I3C) are more potent inducers of CYP gene expression than skatole.

Humans, like other mammal species, are exposed to skatole produced from the intestinal bacterial metabolism of tryptophan [34]. Additionally, smokers are exposed to skatole originating from tryptophan pyrolysis and humans could also be exposed to skatole from various food sources [2, 35]. Moreover, patients with liver diseases may have higher skatole plasma concentrations compared to people with normal liver functions [36]. Additionally, as shown in pigs, dietary changes may favor a skatole-producing microflora in the intestine, resulting in more skatole available for absorption [37–39]. Nevertheless, the basic concentration of skatole in human liver or plasma is unknown. Several old studies could not detect skatole in samples from healthy volunteers. However, analytical techniques have improved since then and now it may be possible to detect skatole in human samples. Moreover, skatole metabolites have been identified in human urine [40], supporting the presence of skatole in human plasma. In the current study, we used skatole concentrations from 1 to 100 μM. The latter concentration is probably much higher than what could be found in normal human liver; however, it could be relevant in some pathological conditions, as high concentrations of skatole metabolites has been found in plasma of schizophrenic patients [41, 42]. Moreover higher skatole amount in feces has been found in patients with colon cancer [43, 44], suggesting higher plasma skatole, as skatole is freely absorbed through the gut wall [45]. Additionally, specific diets can change the gut-flora, increasing skatole production and presence in the gut. In these situations, skatole or its metabolites can act as weak AhR activators, thus inducing the expression of AhR-regulated genes, such as *CYP1A1*, *CYP1A2* and *CYP1B1*.

Previous studies have shown increased *CYP1A1* expression after treatment with skatole and other tryptophan metabolites [19–22, 46]. Specifically, in human normal bronchial epithelial cells, the skatole-induced CYP1A1 mRNA/protein expression increase was dependent on AhR [21], as observed in our study in hepatic cells. In a recently published study, it was shown that skatole treatment induced *CYP1A* mRNA expression in a colon derived human cell model (Caco2) and activated AhR controlled reporter gene in HepG2 [22]. Moreover, we show that skatole effect on CYP1A1 expression could be limited by chemical inhibition of CYP450 activity. This suggests that a skatole metabolite is responsible, at least in part, for AhR activation, or is a more potent AhR activator than skatole. One such metabolite could be I3C, which is produced via CYP450-dependent metabolism of skatole [14, 33, 37]. Accordingly, treatment of HepG2-C3 cells with micromolar concentrations of I3C for 24 h increased *CYP1A1* mRNA expression, as previously reported [47].

Skatole is a weaker AhR activator than TCDD. As observed for other weak AhR activators [48, 49], skatole exhibits an antagonistic effect on TCDD-mediated AhR activation. Indeed, compared to TCDD alone, co-treatment with TCDD and skatole produced a weaker response in reporter gene assays and a lower CYP1A mRNA and protein up-regulation in PHHs, suggesting that skatole is a partial AhR agonist. Moreover, the fact that the inhibitory effect of skatole on TCDD-induced CYP1A expression is maintained in the presence of ABT (an inhibitor of CYP activity) suggests that this inhibition is caused by skatole and not by a metabolite. It was recently described that tryptophan metabolites can exert diverse effects on AhR activity. Tryptamine and indole 3-acetate were AhR agonists, whereas indole was an AhR antagonist that inhibited TCDD-induced *CYP1A1* expression [46].

HepG2-C3 cells exhibit very low CYP450-dependent activity compared to PHHs. This could explain the differences observed between HepG2-C3 cells and PHHs. Indeed, skatole antagonist effect on TCDD-mediated activation of AhR is better maintained in HepG2C3 cells (Fig 4A) than in PHHs (Fig 4C and 4D). Moreover, in PHHs, the antagonistic effect of skatole on TCDD-induced *CYP1A1* mRNA expression was observed only after 8 h of incubation, while it was maintained for 24 h for both CYP1A2 and CYP1B1. This seems surprisingly given the common pathway of induction *via* AhR. In addition, in pTXINV-XRE-luc reporter plasmid-transfected HepG2-C3 cells, co-treatment with skatole and TCDD reduced luciferase activity compared to TCDD alone, suggesting that the antagonistic effect of skatole is at the level of AhR activation. Nevertheless, mRNA expression is not only the result of gene transcription regulation. Indeed, mRNA degradation also influences mRNA content and can affect the specific mRNA expression of genes the transcription of which is controlled by common pathways. Moreover, although *CYP1A1*, *CYP1A2* and *CYP1B1* share AhR as a transcriptional regulator, their tissue-specific expression profiles are very different, suggesting that other transcription factors participate in their regulation. This difference between CYP1A1 and CYP1A2/1B1 needs to be further investigated.

Importantly, skatole was administered in micromolar concentrations, while TCDD concentrations were in the nanomolar range. How skatole can act as an antagonist of TCDD is not elucidated by the current study. They could compete for the same ligand-binding domain on the receptor, as suggested by the antagonist effect of CH223191, which was previously shown to be a pure AhR antagonist that competes with TCDD for binding to AhR [24].

Finally, we observed, that I3C, a skatole metabolite, also exerts a positive effect on AhR activation in HepG2-C3 cells, as previously described (review in [16]). However, differently from skatole, co-incubation with TCDD did not affect TCDD-mediated *CYP1A1* induction, suggesting that I3C, like tryptamine and indole 3-acetate, is an AhR agonist. Dietary administration of I3C protects wild type mice against intestinal cancer development [50] and reduces hepatic steatosis in mice fed a high-fat diet [51]. Skatole and its metabolites may therefore affect liver homeostasis in a complex manner.

**Fig 7:**
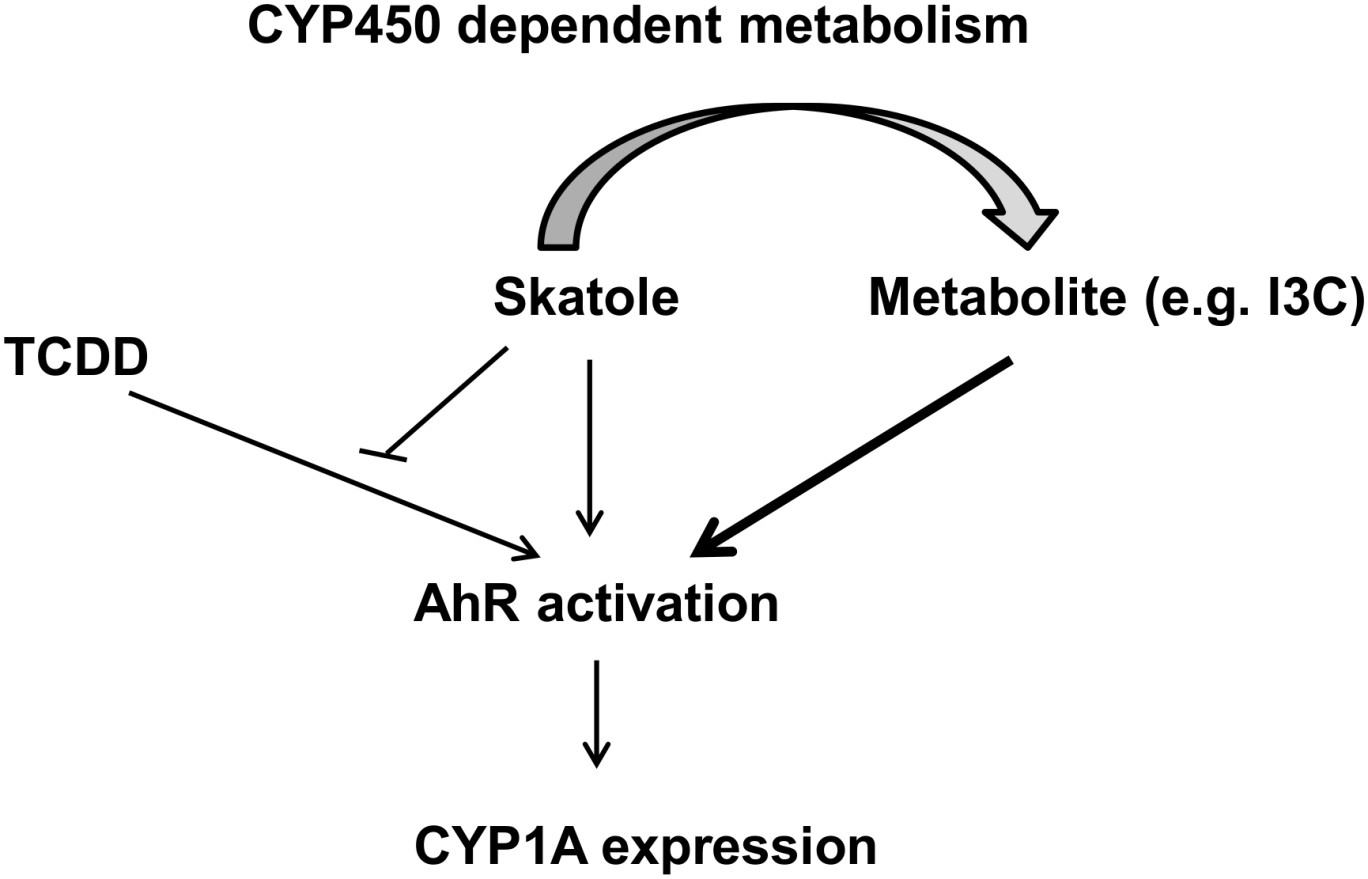
Schematic model of the impact of skatole on AhR activity, as suggested from the findings of this work.

In summary, the present study shows that skatole and some of its metabolites (for instance, I3C) are AhR activators, affecting the expression of AhR-target genes in PHHs. Moreover, skatole acts as an antagonist of TCDD-mediated AhR activation (Fig 7), suggesting that skatole is a partial AhR agonist. Thus, the concentration and ratio of abundance of skatole and its metabolites, alone or in combination with other dietary factors and potential exogenous AhR ligands (i.e., contaminants), can potentially influence liver functions.

## Supporting Information

S1 Fig HepG2-C3 cell viability after Actinomyocin D treatment. (PDF)

S2 Fig Housekeeping genes mRNA expression in HepG2-C3. (PDF)

S3 Fig CYP1A2 mRNA expression in HepG2-C3. (PDF)
