# Supplementary material for: Skatole (3-Methylindole) Is a Partial Aryl Hydrocarbon Receptor Agonist and Induces CYP1A1/2 and CYP1B1 Expression in Primary Human Hepatocytes
Source: PLoS One. 2016 May 3;11(5):e0154629. doi: 10.1371/journal.pone.0154629 (PMC4854444; doi:10.1371/journal.pone.0154629)

S1 figure. HepG2-C3 cell viability after Actinomyocin D treatment

HepG2-C3 cells (n = 3) were treated with 4 µM actinomycin D (ACT) for 9 hours. Following treatment cell viability was assessed using the CellTiterGlo assay (Promega).

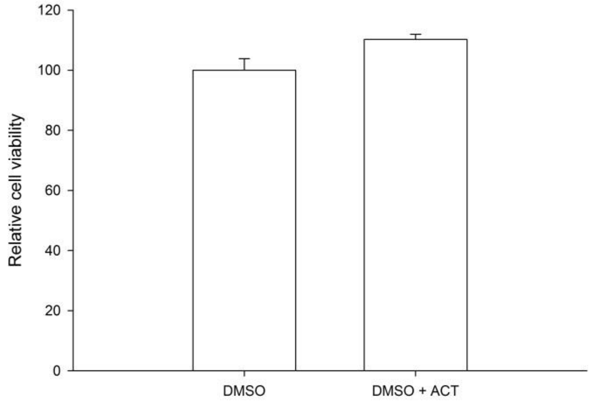

Supplement: S1 Fig — (PDF) [file pone.0154629.s001.pdf]
