# Supplementary material for: Skatole (3-Methylindole) Is a Partial Aryl Hydrocarbon Receptor Agonist and Induces CYP1A1/2 and CYP1B1 Expression in Primary Human Hepatocytes
Source: PLoS One. 2016 May 3;11(5):e0154629. doi: 10.1371/journal.pone.0154629 (PMC4854444; doi:10.1371/journal.pone.0154629)

S3 figure. CYP1A2 mRNA expression in HepG2-C3.

RT-qPCR analysis of CYP1A2 mRNA expression in HepG2-C3 cells following incubation with 4 µM actinomycin D (ACT) for 1 h and incubation with 10 nM TCDD or 10, 50 or 100 µM skatole for 8 h (n = 3).

ND, not detected.

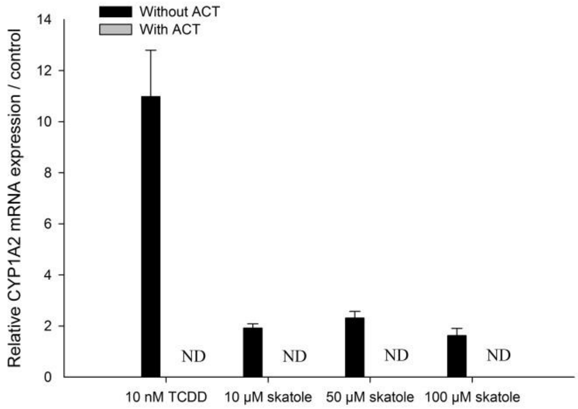

Supplement: S3 Fig — (PDF) [file pone.0154629.s003.pdf]
